# Canine vector-borne diseases in Brazil

**DOI:** 10.1186/1756-3305-1-25

**Published:** 2008-08-08

**Authors:** Filipe Dantas-Torres

**Affiliations:** 1 Departamento de Imunologia, Centro de Pesquisas Aggeu Magalhães, Fundação Oswaldo Cruz, PO Box 7472, Recife, 50670420, Pernambuco, Brazil

## Abstract

Canine vector-borne diseases (CVBDs) are highly prevalent in Brazil and represent a challenge to veterinarians and public health workers, since some diseases are of great zoonotic potential. Dogs are affected by many protozoa (e.g., *Babesia vogeli*, *Leishmania infantum*, and *Trypanosoma cruzi*), bacteria (e.g., *Anaplasma platys *and *Ehrlichia canis*), and helminths (e.g., *Dirofilaria immitis *and *Dipylidium caninum*) that are transmitted by a diverse range of arthropod vectors, including ticks, fleas, lice, triatomines, mosquitoes, tabanids, and phlebotomine sand flies. This article focuses on several aspects (etiology, transmission, distribution, prevalence, risk factors, diagnosis, control, prevention, and public health significance) of CVBDs in Brazil and discusses research gaps to be addressed in future studies.

## Background

Canine vector-borne diseases (CVBDs) constitute an important group of illnesses affecting dogs around the world. These diseases are caused by a diverse range of pathogens, which are transmitted to dogs by different arthropod vectors, including ticks, fleas, lice, triatomines, mosquitoes, tabanids, and phlebotomine sand flies.

CVBDs are historically endemic in tropical and subtropical regions and have increasingly been recognized, not only in traditionally endemic areas, but also in temperate regions [1]. This may be attributed to several factors, including the availability of improved diagnostic tools, higher public awareness about CVBDs, dog population dynamics, and environmental and climate changes [2], which directly influences the distribution of arthropod vectors and the diseases they transmit.

CVBDs have long been recognized in Brazil [3]. At the beginning of the 21st century, CVBDs are prevalent in all regions of the country and some of them have increasingly been recognized in previously free areas, as it is the case of canine leishmaniasis in São Paulo, Southeast Brazil [4-11]. Despite their recognized importance, many aspects concerning epidemiology and public health significance of CVBDs in Brazil are still poorly known and data have not been comprehensively discussed.

This article summarizes several aspects (etiology, transmission, distribution, prevalence, risk factors, diagnosis, control, prevention, and public health significance) of CVBDs in Brazil and discusses research gaps to be addressed in future studies.

## Protozoal diseases

### Canine babesiosis

**Figure 1:**
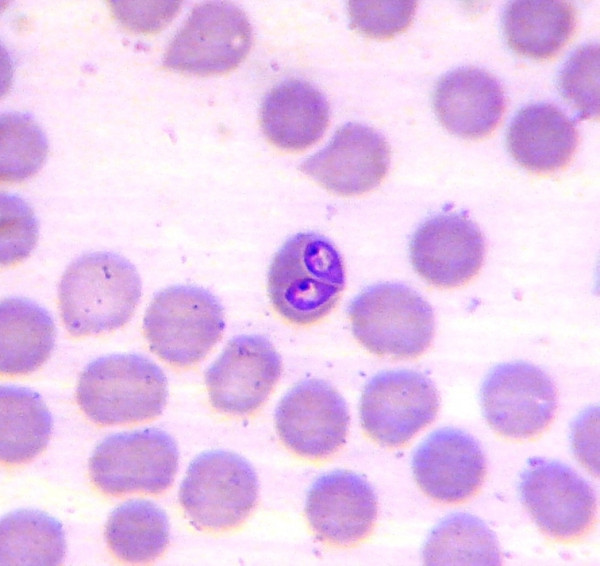
***Babesia vogeli***. Two *Babesia *sp. trophozoites in a blood smear from a naturally infected dog.

**Figure 2:**
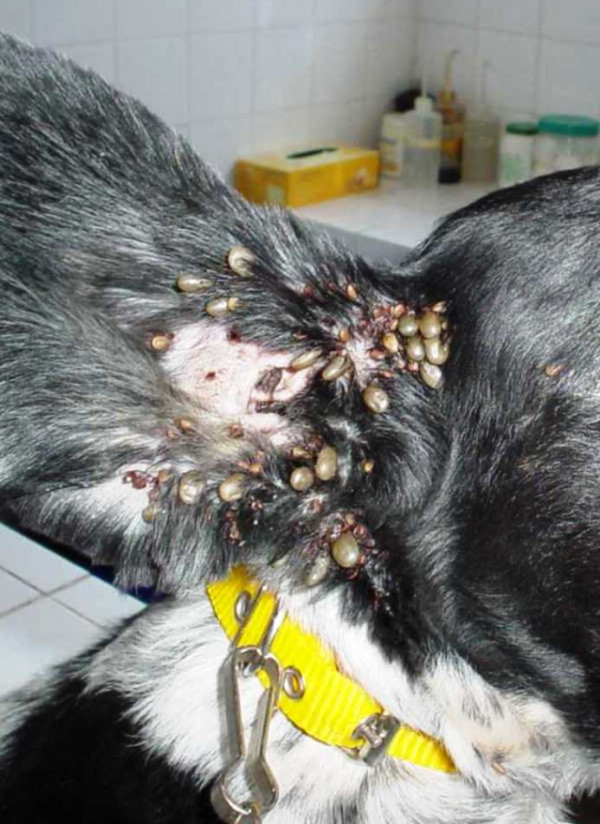
***Rhipicephalus sanguineus***. A dog heavily infested by *Rhipicephalus sanguineus *ticks.

Canine babesiosis has been recognized in Brazil since the beginning of the 20th century [12]. This disease is caused by *Babesia vogeli *(= *Babesia canis vogeli*) (Piroplasmida: Babesiidae) (Fig. 1), which has recently been molecularly characterized in Brazil [13]. Cases of *Babesia gibsoni *infection in Brazilian dogs have also been reported [14]. The only proven vector of *B. vogeli *in Brazil is *Rhipicephalus sanguineus *(Fig. 2), which is also the suspected vector of *B. gibsoni *[15].

Canine babesiosis is prevalent in virtually all Brazilian regions [12,16-24]. The prevalence of infection ranges from 35.7 [24] to 66.9% [16] in serological surveys and from 1.9 [23] to 42% [21] by cytology on blood smears. The incidence of disease seems to be higher among adult dogs [24], although young dogs are also highly susceptible to infection [22]. Apparently, there are no breed or sex predilections [16,21,24-26].

The diagnosis of canine babesiosis is usually based on the presence of suggestive clinical signs (e.g., apathy, fever, anorexia, weigh loss, pale mucous membranes, and jaundice) and patient history. The infection by *Babesia *spp. is confirmed by the examination of Giemsa-stained peripheral blood smears. A detailed review of all aspects, including diagnosis and treatment, of canine babesiosis in Brazil can be found elsewhere [22].

### Canine leishmaniasis

**Figure 3:**
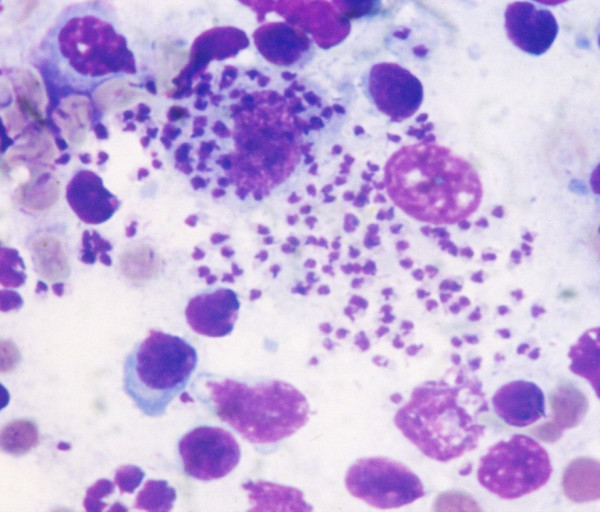
***Leishmania infantum***. Several *Leishmania infantum *amastigotes in a bone marrow smear from a naturally infected dog.

**Figure 4:**
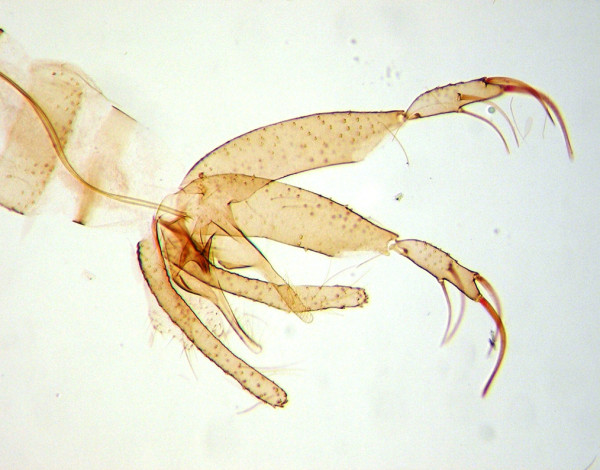
***Lutzomyia whitmani***. External genitalia of a male of *Lutzomyia whitmani*, which contains structures of major taxonomic importance.

Canine leishmaniasis was firstly recognized in Brazil during the 1930s [27]. This disease is mainly caused by *Leishmania infantum *(Kinetoplastida: Trypanosomatidae) (Fig. 3), sometimes referred to as *Leishmania chagasi *or *Leishmania infantum chagasi *[28]. Infection by other *Leishmania *species (e.g., *Leishmania amazonensis*) have also been reported [7,10] and cases of co-infection by two species (e.g., *L. infantum *and *Leishmania braziliensis*) as well [29]. The main vector of *L. infantum *in Brazil is *Lutzomyia longipalpis *(Diptera: Psychodidae). Other modes of transmission, including by *Rh. sanguineus *ticks, are suspected to occur [30,31], particularly in foci where suitable phlebotomine sand fly vectors are absent (e.g., Recife, Northeast Brazil) [32]. The vectors of *L. amazonensis *and *L. braziliensis *vary from region to region and several species may eventually be involved, including *Lutzomyia whitmani *(Fig. 4) and *Lutzomyia intermedia *(reviewed in [33]).

Canine visceral leishmaniasis by *L. infantum *is endemic in all Brazilian regions [34-47], except in South where the disease is seldom recognized [44,48,49]. Canine cutaneous leishmaniasis by *L. braziliensis *is also prevalent in all regions [7,10,38,50-58], except in Center-West. The only two cases of *L. amazonensis *infection in dogs reported so far were diagnosed in Southeast Brazil [10]. The prevalence of *Leishmania *spp. infection in dogs varies widely [38,47,59,60] and may be as high as 67% in highly endemic foci [61]. Risk factors associated with canine leishmaniasis have extensively been studied in Brazil. There appears to be no sex predilection [35,60]. Although the prevalence of infection is often higher among males [47], this seems to be a matter of exposition rather than sex-related susceptibility. The prevalence is also higher in young dogs [47]. Some breeds (e.g., boxer and cocker spaniel) are apparently more susceptible to *L. infantum *infection [60]. Short-furred dogs are at a higher risk of infection [60] and this has been attributed to the fact that their short-hair makes them more exposed to phlebotomine sand fly bites.

**Figure 5:**
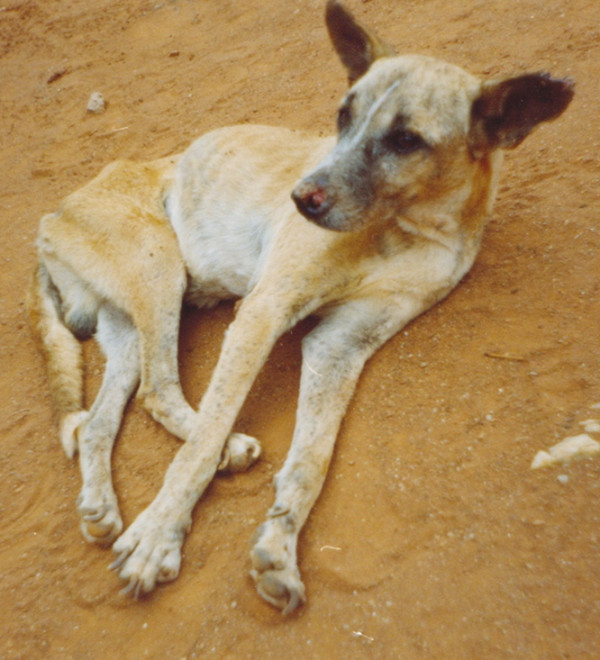
**Canine visceral leishmaniasis**. A dog displaying a typical clinical picture of visceral leishmaniasis.

The diagnosis of canine leishmaniasis is based on the presence of suggestive clinical signs (e.g., weight loss, dermatitis, hair loss, mouth and skin ulcers, enlarged lymph nodes, onychogryphosis, and conjunctivitis) (Fig. 5) and on a positive serological response to *Leishmania *antigens [47,62]. Detailed information on several aspects of canine leishmaniasis, including diagnosis and treatment, can be found elsewhere [31,63,64].

The treatment of canine leishmaniasis is not routinely practiced in Brazil. Until the middle of the 1980s, most attempts to treat Brazilian dogs affected by leishmaniasis were unsuccessful [65]. Nowadays, there is scientific evidence supporting the treatment of canine leishmaniasis in Brazil [66-69]. However, although the available protocols are effective in promoting clinical improvement, a parasitological cure is seldom achieved [66-71]. Hence, considering the importance of dogs in the epidemiology of zoonotic visceral leishmaniasis, the Ministry of Health and the Ministry of Agriculture, Livestock and Food Supply have recently prohibited the treatment of canine visceral leishmaniasis in Brazil [see Addendum].

### Canine hepatozoonosis

**Figure 6:**
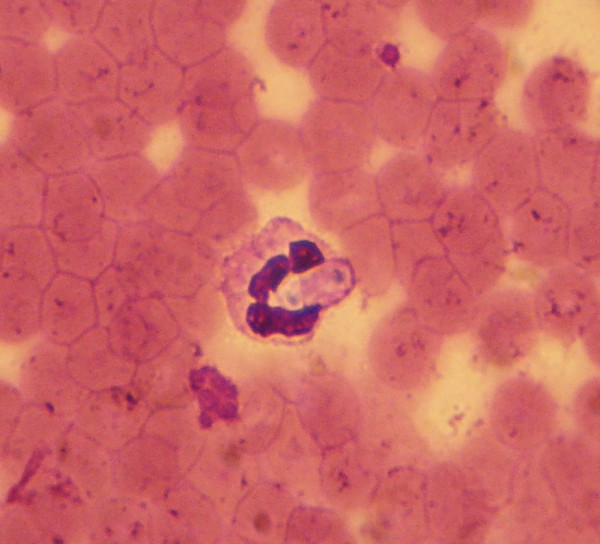
***Hepatozoon canis***. A gamont of *Hepatozoon canis *in a blood smear from a naturally infected dog.

**Figure 7:**
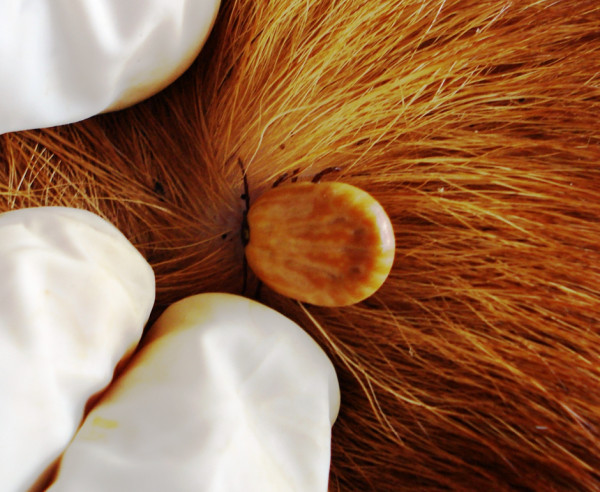
***Amblyomma ovale***. A female of *Amblyomma ovale *firmly attached to and feeding on a dog.

Canine hepatozoonosis was firstly diagnosed in Brazil during the 1970s [72]. This disease is caused by *Hepatozoon canis *(Apicomplexa: Hepatozoidae) (Fig. 6), which has recently been molecularly characterized in Brazil [73-75]. Dogs become infected by ingestion of a tick containing mature *H. canis *oocysts. Ticks involved in the transmission of *H. canis *in Brazil include some *Amblyomma *species, particularly *Amblyomma aureolatum*, *Amblyomma ovale *(Fig. 7), and *Amblyomma cajennense *[76-78]. *Rhipicephalus sanguineus*, which is a known vector of *H. canis *in the Old World, may also play a role in the transmission of this pathogen in Brazil.

Canine hepatozoonosis is prevalent in Center-West, Northeast, South, Southeast [72-82], and much probably in the North region. The prevalence of infection may be as high as 39% in some rural areas [76]. Little is known about the risk factors associated with *H. canis *infection in Brazil. The infection is more prevalent in rural areas [76], where dogs are more exposed to *Amblyomma *ticks. However, this association is not fully understood, because dogs from urban areas are highly exposed to *Rh. sanguineus *[83], a major vector of *H. canis *in the Old World [84].

The diagnosis of canine hepatozoonosis is based on the presence of suggestive clinical signs (e.g., apathy, anorexia, pale mucous membranes, fever, weight loss, diarrhoea, vomit, and muscle pain) and on the observation of *H. canis *gamonts in leucocytes in Giemsa-stained blood smears [79,84-87]; the sensitivity is higher if peripheral blood is used [78]. More information on diagnosis and treatment of canine hepatozoonosis can be found elsewhere [84,86].

### Canine trypanosomiasis

Canine trypanosomiasis has been studied in Brazil since the beginning of the 20th century [88]. This disease is caused by protozoa of the genus *Trypanosoma *(Kinetoplastida: Trypanosomatidae) and has sporadically been recognized in Brazil. *Trypanosoma *species known to infect dogs in Brazil are *Trypanosoma evansi *[89-96], *Trypanosoma cruzi *[97-100], and possibly *Trypanosoma rangeli *[101], the latter species is normally nonpathogenic.

The vectors of *T. cruzi *(a stercorarian species) are triatomines of the genera *Panstrongylus*, *Rhodnius*, and *Triatoma *(Hemiptera: Triatominae). *Rhipicephalus sanguineus *ticks feed on dogs infected by *T. cruzi *can acquire the infection [102], but there is no evidence supporting the development and subsequent transmission to naïve dogs. *Trypanosoma cruzi *infection in dogs is prevalent in all regions, except in South [103]. In areas where American trypanosomiasis (or Chagas disease) is endemic, it is estimated that around 15–50% of the dogs are exposed to *T. cruzi *infection [97-100,104,105]. Clinically, the infection is of minor significance; that is, infected dogs are often asymptomatic carriers. In an experimental model, only sporadic febrile episodes were noted during the first weeks post inoculation [106]. Some dogs developed chronic focal and discrete myocarditis, which was only noticed during necropsy [106].

**Figure 8:**
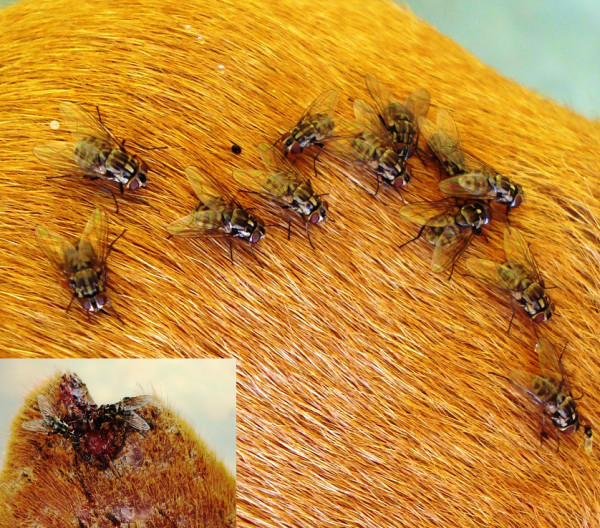
***Stomoxys calcitrans***. Several stable flies (*Stomoxys calcitrans*) feeding on a dog.

The vectors of *T. evansi *(a salivarian species) are hematophagous flies of the genera *Tabanus *(Diptera: Tabanidae) and *Stomoxys *(Diptera: Muscidae) (Fig. 8). *Trypanosoma evansi *infection in dogs is found predominately in Center-West and South regions [89-96,107,108]. In Mato Grosso (Center-West Brazil), for instance, the prevalence of *T. evansi *infection is serologically estimated to be around 30% [90]. Dogs are regarded as efficient reservoirs of *T. evansi*, which is the causative agent of a severe disease affecting horses, commonly known as *mal de cadeiras *or *surra*. The infection in dogs is also severe and potentially fatal [93]. Clinical signs include edema of the hind limbs, anorexia, apathy, dehydration, pale mucous membranes, fever, and weight loss [93,108-110].

Vectors of *T. rangeli *are triatomines of the genus *Rodnius*. While *T. cruzi *is transmitted through the feces of triatomines, *T. rangeli *is can be transmitted through both feces and saliva. *Trypanosoma rangeli *is widely spread in Brazil and has been found on a large number of hosts, including marsupials, rodents, and humans [101,111-114]. While nonpathogenic neither to dogs nor to humans, *T. rangeli *can be confounded with *T. cruzi*, which poses a challenge for the diagnosis of Chagas diseases, particularly in areas where both species are endemic. The distinction between *T. rangeli *and *T. cruzi *can be done by several biological, immunological, biochemical and molecular assays. The characteristic biological behavior in the invertebrate host is considered the best method for their differentiation [115].

### Nambiuvú

**Figure 9:**
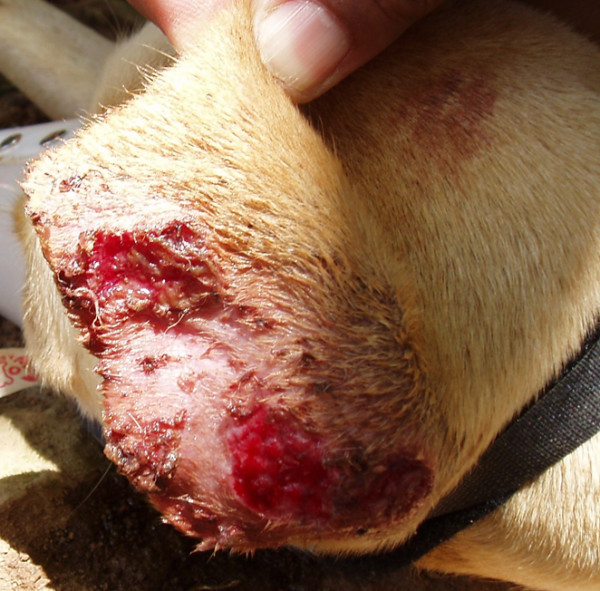
**A dog with clinical signs of the so-called *Nambiuvú***. Massive bleeding from the skin covering the dorsal surface of the pinna.

*Nambiuvú *(in English, bloody ears) or *peste de sangue *(bleeding plague) was firstly recognized in Brazil in 1908 [116]. This little known disease is caused by *Rangelia vitalli *(Piroplasmorida), a protozoan whose current taxonomic position is uncertain. The infection is thought to be transmitted by ticks [117]. Cases of *Nambiuvú *have been recognized in Center-West, South, and Southeast regions [117-120]. The diagnosis of *Nambiuvú *is based on the presence of suggestive clinical signs (e.g., anemia, jaundice, fever, splenomegaly, and persistent bleeding from the nose, oral cavity, and tips, margins and outer surface of the pinnae) (Fig. 9) and on the observation of the parasites within endothelial cells of blood capillaries in necropsy samples. Recent information on several aspects of *Nambiuvú *can be found elsewhere [117,121].

## Bacterial diseases

### Canine monocytic ehrlichiosis

**Figure 10:**
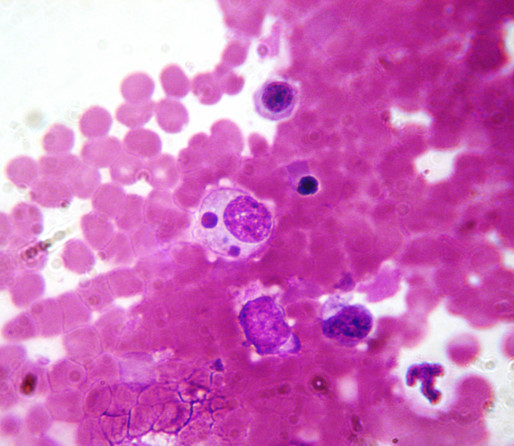
***Ehrlichia canis***. A morula of *Ehrlichia canis *in a bone marrow smear from a naturally infected dog.

Canine monocytic ehrlichiosis was firstly recognized in Brazil in the 1970s [122]. This disease is caused by *Ehrlichia canis *(Rickettsiales: Anaplasmataceae) (Fig. 10), which was firstly isolated in Brazil in 2002 [123]. The agent of canine monocytic ehrlichiosis is well characterized in Brazil [124-128], where it is transmitted by *Rh. sanguineus *[124]. Other *Ehrlichia *species found in Brazil – e.g., *Ehrlichia chaffeensis*; [129] – are also suspected to infect dogs. In fact, there is serological evidence of *E. chaffeensis *infection in Brazilian dogs [130].

Canine ehrlichiosis is prevalent in virtually all regions of Brazil [24,124-127,131,132]. This disease affects around 20–30% of the dogs referred to veterinary clinics and hospitals in Brazil [24,124,131], but the prevalence of infection vary widely from region to region [23,76,126,128,131-135]. The prevalence of infection can be as high as 46.7% in asymptomatic [128] and 78% in symptomatic dogs [132]. The risk of *E. canis *infection is higher for dogs that live in houses when compared to dogs living in apartments [23]. This is expected because dogs that live in houses with backyards are theoretically more exposed to ticks than those living in apartments. Seroepidemiological studies revealed that male adult dogs are more likely to present antibodies to *E. canis*, particularly those infested by ticks [24,134].

The diagnosis of canine ehrlichiosis is usually based on clinical signs (e.g., fever, pale mucous membranes, apathy, anorexia, lymphnode enlargement, and weight loss) and on the observation of *E. canis *morulae in Giemsa-stained peripheral blood smears. More information on diagnosis and treatment of canine ehrlichiosis can be found elsewhere [136].

### Canine anaplasmosis

Canine anaplasmosis is caused by *Anaplasma platys *(formerly *Ehrlichia platys*) (Rickettsiales: Anaplasmataceae) and has been recognized sporadically in Brazil. There are different *A. platys *strains circulating in Brazilian dogs, as revealed by analysis of partial sequences of the 16S rRNA gene [137]. The vector of *A. platys *is still unknown or unproven. Ticks of various genera (e.g., *Rhipicephalus*, *Dermacentor*, and *Ixodes*) have been found naturally infected by *A. platys *around the world [138-142]. The suspected vector of *A. platys *in Brazil is *Rh. sanguineus*.

Canine anaplasmosis has been found in all regions of Brazil, although few cases have been formally published in the literature [124,127,143-145]. The prevalence of *A. platys *infection ranges from 10.3 [146] to 18.8% [145]. Little is known about risk factors associated with canine anaplasmosis in Brazil. The infection by *A. platys *is seldom associated with clinical disease, except in cases of co-infection with other organisms (e.g., *E. canis *and *B. vogeli*), which is common in Brazil [19,21,127,134]. Typically, dogs infected by *A. platys *display only a cyclic thrombocytopenia, but no hemorrhagic events are noted. The laboratory diagnosis is based on the observation of *A. platys *inclusions in platelets in peripheral blood smears stained with ordinary hematological staining methods. Serological studies have never been performed and molecular techniques are currently restricted to research.

### Canine Rocky Mountain spotted fever

**Figure 11:**
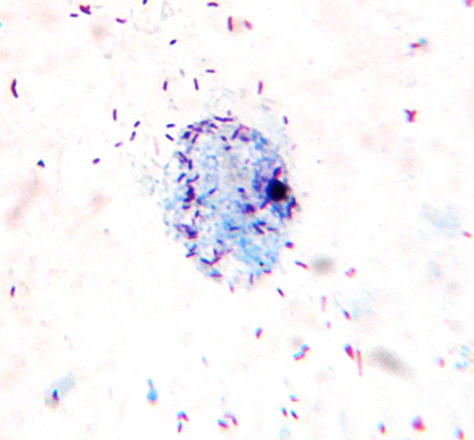
***Rickettsia rickettsii***. *Rickettsia rickettsii *growing in Vero cells.

**Figure 12:**
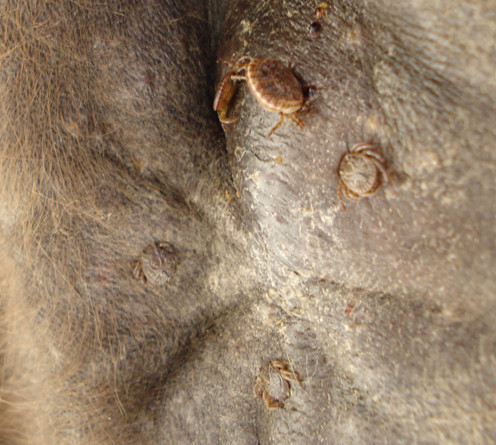
***Amblyomma cajennense***. *Amblyomma cajennense *ticks feeding on a horse.

Canine Rocky Mountain spotted fever is caused by *Rickettsia rickettsii *(Fig. 11) and has been associated with significant morbidity and occasional mortality in the United States [147,148]. Serological surveys conducted in Brazil have shown that dogs from some Rocky Mountain spotted fever-endemic areas (e.g., Minas Gerais and São Paulo) are exposed to *R. rickettsii *infection [129,149-154]. The vectors of *R. rickettsii *are *Amblyomma *ticks, mainly *Am. cajennense *[155] (Fig. 12) and *Am. aureolatum *[156]. Additionally, *Rh. sanguineus *ticks have the potential to be involved in the *R. rickettsii *transmission cycle in areas other than Mexico and United States, including Brazil [157]. Serological surveys in Minas Gerais, Espírito Santo, Rondônia, and São Paulo revealed that the prevalence of anti-*R. rickettsii *antibodies in dogs ranges from 4.1 to 64% [129,149-154,158]. However, it is difficult to estimate the actual prevalence of *R. rickettsii *infection in dogs using serological tests, because of their low specificity [157].

Little is known about the risk factors associated with *R. rickettsii *infection in Brazilian dogs. In a study conducted in São Paulo, the proportion of dogs positive to anti-*R. rickettsii *antibodies increased with age [158]. Although there is no information about clinical cases of Rocky Mountain spotted fever in dogs in Brazil, veterinarians working in areas where human cases have been reported must consider the possibility of this disease to request laboratory tests that will allow a proper diagnosis.

### Canine haemobartonellosis

Canine haemobartonellosis has been sporadically recognized in Brazil, but little is known about this disease in this country, because few reports have been formally published in the literature. This disease is caused by *Mycoplasma haemocanis *(formerly *Haemobartonella canis*) (Mycoplasmatales: Mycoplasmataceae), which is transmitted by *Rh. sanguineus *[159]. *Mycoplasma haemocanis *infection in dogs has been recognized in South and Southeast Brazil [17,144,160-162]. Clinical disease in immunocompetent animals is uncommon. On the other hand, immunosuppressed dogs (e.g., splenectomized dogs) are particularly susceptible to infection [161,163].

Clinical signs include pale mucous membrane, weight loss, apathy, anorexia, and fever [164]. The diagnosis of *M. haemocanis *infection is based on microscopic examination of blood smears stained with ordinary hematological staining techniques. Serological and molecular assays have also been used [164].

### Canine borreliosis

A Lyme-like illness has been recognized in humans in Brazil since 1989 [165], although the true identity of the causative agent has not yet been determined. Serological surveys conducted in Southeast Brazil confirmed that dogs are often exposed to infection by *Borrelia burgdorferi *(*sensu lato*). *Borrelia*-like spirochetes have been detected in *Ixodes *ticks in the State of São Paulo [166], but the possible vectors of *B. burgdorferi s. l*. in Brazil are largely unknown. *Amblyomma *ticks are also suspected to be involved in transmission [167].

The prevalence of anti-*B. burgdorferi s. l*. antibodies in Brazilian dogs ranges from less than 1 up to 20% [130,132,168,169]. The infection in dogs is usually asymptomatic and there appears to be no correlation between seropositivity and sex or age of the animals [169]. As expected, the seropositivity correlates with history of previous contact with ticks [169]. At present, there is no information about the treatment of dogs with suspected *B. burgdorferi s. l*. infection in Brazil.

## Helminthiasis (heartworm and tapeworm)

### Canine dirofilariasis

**Figure 13:**
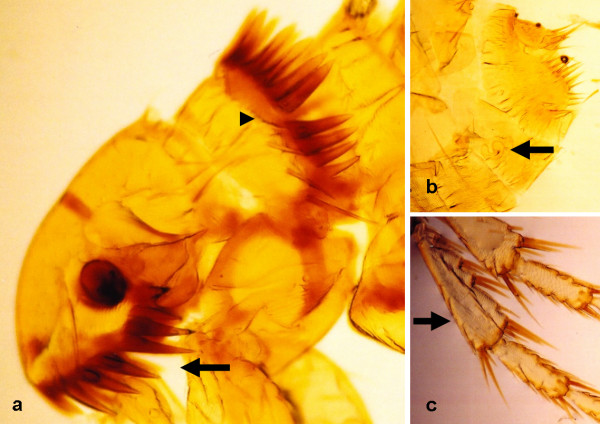
***Ctenocephalides felis *female**. (a) Flea's head, exhibiting the characteristic genal (arrow) and pronotal (arrowhead) combs. (b) Spermatheca (arrow). (c) Chaetotaxy of tibia (arrow) of leg III.

Canine heartworm was firstly recognized in Brazil in 1878 [3]. The disease is caused by *Dirofilaria immitis *(Nematoda: Onchocercidae), which is transmitted by many mosquito species. *Aedes scapularis *and *Aedes taeniorhynchus *are implicated as the primary vectors, while *Culex quinquefasciatus *is a secondary vector [170-174]. Another filarid nematode commonly found infecting dogs in Brazil is *Acanthocheilonema reconditum *(formerly *Dipetalonema reconditum*) (Nematoda: Onchocercidae), whose intermediate hosts are fleas (*Ctenocephalides canis *and *Ctenocephalides felis*) (Fig. 13) and lice (*Heterodoxus spiniger *and *Trichodectes canis*) [175,176]. *Acanthocheilonema reconditum *infection usually causes no clinical signs in dogs. Despite this, it is important to distinguish the microfilaria of *A. reconditum *from that of *D. immitis*, as these filarid nematodes are often found in sympatry.

*Dirofilaria immitis *is prevalent in virtually all regions of Brazil [3,172,177-185]. The prevalence of *D. immitis *infection in dogs varies widely and can be higher than 60% in highly endemic foci [185]. The countrywide prevalence has decreased from 7.9% in 1988 to 2% in 2001 [186]. The possible reasons for this decrease include the reduction of transmission as a result of effective chemoprophylaxis and/or reduction of microfilaremic dog populations due to the off-label use of injectable ivermectin [187]. The risk of *D. immitis *infection is grater in dogs living in coastal regions [170,172,182,187] and in dogs older than two years [185]. Apparently there is no sex or breed predisposition [172,182]. In some areas, the prevalence of infection is higher among males [177,185], although this is likely to be a matter of exposure rather than sex-related susceptibility. Likewise, the prevalence of infection seems to be higher among mixed-breed dogs [188].

The diagnosis of canine heartworm is based on clinical signs (e.g., coughing, exercise intolerance, dyspnea, weight loss, cyanosis, hemoptysis, syncope, epistaxis, and ascites). The infection is confirmed by the observation of microfilariae in blood samples using the modified Knott's test or the detection of antigens produced by adult heartworms using commercial enzyme-linked immunosorbent assay kits [189].

### Dipylidiasis (tapeworm infection)

Dipylidiasis is caused by *Dipylidium caninum *(Cestoda: Dipylidiidae), whose intermediate hosts include fleas (*C. felis *and *C. canis*) and lice (*T. canis *and *H. spiniger*). Dogs become infected by ingestion of intermediate hosts containing infective cysticercoids (*i. e*., the adult tapeworm encysted in the intestinal wall of an intermediate host) [190]. In a recent study on endosymbionts of *C. felis felis *collected from dogs in Minas Gerais, of 1,500 fleas examined, six (0.4%) were infested by *D. caninum *[191]. Not surprisingly, the infestation by *D. caninum *in dogs (and also in cats) is commonly found in all regions of Brazil [192-198]. The infestation is usually asymptomatic. Some dogs may be seen scooting or dragging the rear end across the floor. This behavior is a consequence of the intense perianal pruritus caused by the rice grain-like proglottids, which can be eventually seen crawling around the anus.

## Control and prevention of CVBDs in Brazil

### Vaccination

At present, only two CVBDs are preventable by vaccination in Brazil. A vaccine (Leishmune, Fort Dodge Animal Health Brazil) against canine visceral leishmaniasis was recently licensed in Brazil [199]. This vaccine is only recommended for healthy, seronegative dogs at the minimum age of four months. The vaccine is well tolerated, although some dogs display transient mild adverse events (e.g., pain, anorexia, apathy, local swelling reactions, vomit, and diarrhea) [200]. Its efficacy is around 80% [43]. However, it is important to state that this vaccine protects dogs against the disease (*i. e*., appearance of clinical signs), but not against *L. infantum *infection [199].

Until recently, there was no vaccine against canine babesiosis in Brazil [22]. A vaccine (Nobivac^® ^Piro, Intervet Brazil) was recently licensed for commercialization in Brazil, but no information about efficacy and safety of this vaccine in preventing canine babesiosis in Brazil is currently available.

### Chemoprophylaxis

The chemoprophylaxis of canine heartworm is usually undertaken in Brazil, using different microfilaricides, such as ivermectin, milbemycin oxime, and selamectin [189]. The chemoprophylaxis of canine babesiosis has been recommended in Brazil [22]. Imidocarb can protect dogs from *B. canis *infection for 2–6 weeks [201], whereas doxycycline is effective in preventing clinical disease, but not infection [202].

### Vector control

Vector control is the only effective measure for the control of most CVBDs in Brazil. The strategies currently used for the control of ticks in Brazil have recently been reviewed elsewhere [22,203]. The control of vectors other than ticks (*i. e*., fleas, lice, mosquitoes, triatomines, and phlebotomine sand flies) is performed by using insecticides under different formulations (pour-on, spot on, spray, *etc*.). The use of insecticide-impregnated collars limits the exposure of dogs to phlebotomine sand flies. However, it has been demonstrated that the impact of such intervention is dependent on collar coverage and loss rate [204]. Moreover, experience shows that this approach is of limited impact, mainly because most dog owners living in endemic areas cannot afford the costs such collars.

### Other control measures

While not universally accepted, the culling of dogs positive to anti-*Leishmania *antibodies is still practiced in Brazil [70,72]. This control measure has been subject of intense, ongoing debate in Brazil. Many dog owners, veterinarians, and non-governmental organizations have opposed the culling of seropositive dogs, both for ethical reasons and due to the lack of scientific evidence supporting the effectiveness of this strategy.

From 1990 to 1994, more than 4.5 million dogs were screened and more than 80,000 were culled in Brazil [205]. In the same period, there was an increase of almost 100% in the incidence of human visceral leishmaniasis [205]. Actually, China is probably the only country where the culling of seropositive dogs seems to have been effective [206]. The possible reasons for the failure of the culling of seropositive dogs in Brazil include: high incidence of infection, limited sensitivity and specificity of available diagnostic methods, the time delays between diagnosis and culling, rapid replacement of culled dogs by susceptible puppies or already infected dogs, and owner's unwillingness to give up asymptomatic seropositive dogs [11,70,206,207]. A recent study conducted in Southeast Brazil suggests that the dog culling as a control measure for human visceral leishmaniasis in Brazil should be re-evaluated [11].

## CVBDs from the public health standpoint

CVBDs constitute a group of diseases of great interest because some vector-borne pathogens affecting dogs in Brazil (e.g., *L. infantum*,*T. cruzi*, and *E. canis*) are potentially zoonotic (see Tables 1, 2, and 3). Despite this, in some instances, there is little research-based evidence supporting the role of dogs in the transmission to these pathogens to humans in Brazil.

**Table 1:** Vector-borne protozoa affecting dogs in Brazil.

Agent	Vector(s)	Distribution ^a^	Zoonotic potential
*Babesia vogeli*	*Rhipicephalus* * sanguineus*	Center-West, North, Northeast, South, Southeast	Yes (but low)
*Babesia gibsoni*	*Rh. sanguineus*?	Southeast, South	No
*Hepatozoon canis*	*Amblyomma *spp., *Rh. * *sanguineus*	Center-West, Northeast, South, Southeast	No
*Leishmania * *amazonensis*	*Lutzomyia *spp.	Southeast	Yes ^b^
*Leishmania * *braziliensis*	*Lutzomyia *spp.	North, Northeast, South, Southeast,	Yes ^b^
*Leishmania infantum*	*Lutzomyia * *longipalpis*, *Lutzomyia *spp.	Center-West, North, Northeast, South, Southeast	Yes
*Rangelia vitalli*	*Amblyomma *spp.?, *Rh. sanguineus*?	Center-West, South, Southeast	No
*Trypanosoma cruzi*	*Panstrongylus *spp., *Triatoma *spp., *Rhodnius *spp.	Center-West, North, Northeast, South, Southeast	Yes
*Trypanosoma evansi*	*Tabanus *spp., *Stomoxys *spp.	Center-West, South	No

^a ^Includes some reports not formally published.

^b ^Dogs are unlikely to be important reservoir hosts for human infection.

**Table 2:** Vector-borne bacteria affecting dogs in Brazil.

Agent	Vector(s)	Distribution ^a^	Zoonotic potential
*Anaplasma platys*	*Rhipicephalus* * sanguineus*?	Center-West, North, Northeast, South, Southeast	Yes (but low)
*Borrelia burgdorferi s.l*.	*Amblyomma *spp.?, *Rh. sanguineus*?	Center-West, Northeast, Southeast	Yes ^b^
*Ehrlichia canis*	*Rh. sanguineus*	Center-West, North, Northeast, South, Southeast	Yes
*Mycoplasma haemocanis*	*Rh. sanguineus*	South, Southeast	No
*Rickettsia rickettsii*	*Amblyomma *spp.,* Rh* *. sanguineus*?	Southeast	Yes ^b^

^a ^Includes some reports not formally published.

^b ^Dogs are unlikely to be important reservoir hosts for human infection.

**Table 3:** Vector-borne helminths affecting dogs in Brazil.

Agent	Vector(s)	Distribution ^a^	Zoonotic potential
*Acanthocheilonema reconditum*	*Ctenocephalides *spp., *Heterodoxus spiniger*, *Trichodectes canis*	Center-West, Northeast, South, Southeast	Yes (but low)
*Dipylidium caninum*	*Ctenocephalides *spp., *H. spiniger*, *T. canis*	Center-West, North, Northeast, South, Southeast	Yes
*Dirofilaria immitis*	*Aedes *spp., *Culex* spp.	Center-West, North, Northeast, South, Southeast	Yes

^a ^Includes some reports not formally published.

Dogs are implicated as important reservoirs of *L. infantum *in Brazil [206-211]. It is interesting to note that in some areas a high proportion of dogs are exposed to *L. infantum *infection [47], but human cases of visceral leishmaniasis are only sporadically notified [210]. In these areas, the low incidence of visceral leishmaniasis may be because of the difficulties in diagnosing and notifying the human cases [207,210], but it also indicate that the role of dogs in the epidemiology of visceral leishmaniasis may vary from region to region [211].

Near a century after its discovery, Chagas disease is still a serious public health concern in Brazil. Dogs are considered to be an efficient source of *T. cruzi *infection and are thought to play a role in the peridomestic transmission cycle [212,213]. However, Southern Cone countries (e.g., Brazil) have experienced significant changes in the epidemiology of Chagas disease in recent years [214]. New studies to understand the current role of dogs in the cycle of transmission of *T. cruzi *in Brazil are needed.

Human ehrlichiosis is an emerging zoonosis that has been suspected to occur in Brazil since 2004 [215,216]. The suspected causative agent is *E. chaffeensis *[216], but tick vectors are completely unknown. Cases of natural infection by *E. chaffeensis *in dogs are suspected to occur in Brazil [129], but this has not yet been confirmed [126]. Cases of human ehrlichiosis caused by *E. canis *infection have been reported in Venezuela [217]. This raises a number of questions about the risk of *E. canis *infection in humans in Brazil as the main vector (*i. e*., *Rh. sanguineus*) of this rickettsial agent is already known to parasitize humans in this country [218,219]. Further molecular studies are urgently needed to characterize the cases of human ehrlichiosis in Brazil.

Human pulmonary dirofilariasis, a zoonosis that has been diagnosed in Brazil since 1887 [220], has been reported in Rio de Janeiro, São Paulo, and Santa Catarina [179,220-227], where the prevalence of *D. immitis *infection in dogs is moderate to high [183,186]. Cases of human dipylidiasis have also been reported in Brazil [228-230]. Dogs play a major role in the transmission of *D. caninum *for humans, and thus must be periodically evaluated for the presence of gastrointestinal helminths and treated accordingly.

Little is known about human babesiosis in Brazil, where clinical cases of are seldom recognized [231-233]. As *B. canis *is rarely involved in cases of babesiosis in humans [234], dogs are unlikely to play a role in the epidemiology of human babesiosis in Brazil. Although dogs are also unlikely reservoirs of *R. rickettsii *[157], they may play a role in bringing ticks to human dwellings, particularly if ticks like *Am. aureolatum *and *Rh. sanguineus *are involved in the transmission.

## Research gaps

*Rhipicephalus sanguineus *is potentially involved in the transmission of at least nine pathogens affecting dogs in Brazil. Despite this, little is known of the relationship between the ecology of *Rh. sanguineus *and the dynamics of CVBDs in Brazil. Further research is needed to clarify the role of *Rh. sanguineus *in the transmission of *A. plays*, *B. gibsoni*, *H. canis*, *R. rickettsii*, and *L. infantum *in Brazil.

Considering that dogs and humans live in close contact and that both dogs and humans are susceptible to infection by *L. infantum *and *L. braziliensis*, it is reasonable to imagine that in areas where dogs are exposed to these pathogens, humans are exposed as well. However, the finding of a dog infected by a given *Leishmania *species should be analyzed carefully to avoid misinterpretation. While the role of dogs in *L. infantum *transmission is well known, their role as reservoirs of other *Leishmania *species is probably minor [208]. The epidemiology of the leishmaniases is complex and varies from region to region and even within each region. The pattern of transmission of *Leishmania *parasites is intimately linked to the behavior of hosts and vectors involved. Local studies are crucial to understand the dynamics of transmission and to provide information for the establishment of vector control programs.

Most information on CVBDs in Brazil has been informally presented in scientific meetings, which makes it difficult to access the actual distribution and prevalence of these diseases across the different geographical regions of the country. For instance, only five CVBDs have been formally reported to occur in the North region, while 13 CVBDs have been recognized in Southeast Brazil. Indeed, this situation reflects the limited number of studies on CVBDs carried out in North in comparison with Southeast Brazil, where there is a large number of researchers working in this field. Further studies to access the countrywide distribution and prevalence of CVBDs should be encouraged. It is also important to evaluate the impact of environmental changes and human behavior on the prevalence and zoonotic potential of CVBDs in Brazil. CVBDs are likely influenced by climate variations and environmental changes. Also, the zoonotic potential of these diseases is probably greater in remote areas where the access to education and healthcare services is limited.

Co-infection by vector-borne pathogens is a common condition among Brazilian dogs [19,21,29,94,127,134,235]. This is expected because these pathogens often share the same arthropod vector. The occurrence of mixed infections is of great practical importance. Just to give an example, the use of serological tests with low specificity to access *L. infantum *infection may lead to an unnecessary culling of dogs infected by *L. braziliensis *or even by *T. cruzi *[236,237], in areas where both species occur. The use of contemporary techniques to distinguish the species of *Leishmania *infecting dogs [7] is highly desirable, particularly where *L. infantum *and *L. braziliensis *occur in sympatry. The burden of co-infections in Brazilian dogs should be investigated and better molecular tools should be developed to improve the accuracy of the diagnosis.

## Conclusion

In this review, it became clear that CVBDs in Brazil should be faced as a priority by public health authorities. Certain vector-borne pathogens infecting dogs in Brazil are of great significance for human health, as it is the case of *L. infantum *and *T. cruzi*. In this scenario, veterinarians play a key role in providing information to owners about what they should do to reduce the risk of infection by zoonotic vector-borne pathogens in their dogs and in themselves.

CVBDs are prevalent in all geographical regions of Brazil and have been increasingly recognized in recent years. In part, this is a result of the improvements achieved in terms of diagnostic tools. On the other hand, factors such as deforestation, rapid urbanization, climate changes, and the indiscriminate use of chemicals may cause a significant impact on the dispersion of arthropod vectors and on the incidence of CVBDs. The impact of such factors on CVBDs in Brazil has not yet been fully addressed and deserves further research.

Today, the use of molecular biology techniques is contributing to the knowledge on the etiology and epidemiology of CVBDs in Brazil. A better understanding about the ecology of the arthropods involved in the transmission of pathogens to dogs in Brazil is essential to reduce the burden of CVBDs, whose magnitude is probably much greater than is actually recognized.

## Addendum

After this manuscript was submitted, the Ministry of Health and the Ministry of Agriculture, Livestock and Food Supply have published an ordinance prohibiting the treatment of canine leishmaniasis in Brazil [238]. Indeed, this ordinance will enhance the debate around the treatment of canine leishmaniasis in Brazil, in the years to come.

## Note added in proof

After the provisional PDF of this review was available, Dr. Michele Trotta (Laboratorio d’Analisi Veterinarie “San Marco,” Padova, Italy) asked me whether there are cases of canine bartonellosis in Brazil. Cases of *Bartonella* spp. infection in dogs have been reported worldwide. It was, however, only recently that antibodies to and DNA of  *Bartonella henselae* and *Bartonella vinsonii* subspecies *berkhoffii* were detected in dogs from Southeast Brazil [132,239]. Further studies are needed to assess the clinical and zoonotic significance of *Bartonella* spp. infection in dogs from different Brazilian regions.

## Competing interests

The author declares that they have no competing interests.
